# Internet of Medical Things Systems Review: Insights into Non-Functional Factors

**DOI:** 10.3390/s25092795

**Published:** 2025-04-29

**Authors:** Giovanni Donato Gallo, Daniela Micucci

**Affiliations:** Department of Informatics, Systems and Communication, University of Milano-Bicocca, 20126 Milan, Italy; daniela.micucci@unimib.it

**Keywords:** IoMT, interoperability, security, self-adaptation, sustainability, configurability, non-functional requirement

## Abstract

Internet of Medical Things (IoMT) is a rapidly evolving field with the potential to bring significant changes to healthcare. While several surveys have examined the structure and operation of these systems, critical aspects such as *interoperability*, *sustainability*, *security*, *runtime self-adaptation*, and *configurability* are sometimes overlooked. Interoperability is essential for integrating data from various devices and platforms to provide a comprehensive view of a patient’s health. Sustainability addresses the environmental impact of IoMT technologies, crucial in the context of green computing. Security ensures the protection of sensitive patient data from breaches and manipulation. Runtime self-adaptation allows systems to adjust to changing patient conditions and environments. Configurability enables IoMT frameworks to monitor diverse patient conditions and manage different treatment paths. This article reviews current techniques addressing these aspects and highlights areas requiring further research.

## 1. Introduction

Internet of Medical Things (IoMT) represents a significant technological advancement in healthcare, characterized by the integration of medical devices with healthcare IT systems through computer networks. This convergence enables the seamless flow of data between remote devices and central systems, enhancing the ability of healthcare providers to monitor, diagnose, and treat patients more effectively.

IoMT includes a wide range of devices, such as wearable fitness trackers, personal health devices (PHDs), and smart medical implants. These devices gather health data from patients (e.g., heart rate, blood pressure, and activity levels) and transmit them in real-time to cloud platforms for storage and analysis, aiding healthcare providers in making well-informed decisions about patient care.

Existing IoMT surveys primarily focus on the functional aspects of IoMT solutions, exploring how these systems enhance patient monitoring, diagnosis, and treatment. Many studies classify IoMT systems based on their architectural structure, such as the perception, network, and application layers. The perception layer includes sensors and wearable devices for data collection, the network layer manages communication and data transmission, and the application layer handles data processing and delivery of medical services to end users [1].

While these functional and architectural classifications are essential for understanding how IoMT systems operate and integrate within healthcare infrastructures, they often overlook non-functional aspects that are equally critical for the successful deployment and operation of IoMT solutions. Factors such as operational efficiency, security, and runtime adaptability, which influence the performance and reliability of IoMT systems, tend to receive less focus in these surveys. This highlights the need for comprehensive studies that also address non-functional aspects, ensuring that IoMT solutions are not only effective but also secure, efficient, and adaptable to the dynamic healthcare environment.

The aim of the article is to review the current literature on IoMT systems, with a focus on the non-functional requirements critical for their effective implementation. Specifically, it examines strategies to ensure that IoMT solutions (1) guarantee *interoperability*, (2) implement mechanisms for *sustainability*, (3) adopt practices for *security*, (4) achieve *runtime self-adaptation*, and (5) provide *configurability* to align with medical needs and available technological solutions. These key aspects are fundamental in IoMT systems, as they directly influence their ability to function effectively in dynamic healthcare environments. Each of these characteristics is detailed below to highlight its importance and role in IoMT implementations.

*Interoperability.* eHealth is a constantly evolving domain. Thus, ensuring seamless interoperability between different systems is essential for improving healthcare delivery and patient outcomes. Interoperability in eHealth refers to the ability of different healthcare information systems, devices, and applications to access, exchange, interpret, and use data cohesively and effectively. This capability is vital for creating an integrated healthcare environment where data flows seamlessly across systems, enabling healthcare providers to access comprehensive and accurate patient information.

*Sustainability.* Ensuring sustainable practices not only reduces the environmental impact of IoMT technologies but also enhances their long-term viability and cost-effectiveness. Energy-efficient designs, the reuse of devices, and responsible lifecycle management are essential to mitigate the ecological footprint of IoMT devices and to contribute to the overall resilience and reliability of healthcare infrastructures.

*Security.* Ensuring robust security measures in IoMT systems is crucial to protect sensitive patient data and maintain trust in healthcare technologies. Given the interconnected nature of these systems, vulnerabilities can expose personal health information to breaches and disrupt critical medical services. Implementing advanced encryption protocols, secure authentication mechanisms, and continuous monitoring for threats not only safeguards privacy but also ensures the uninterrupted functionality of IoMT devices, thereby supporting the reliability and effectiveness of healthcare delivery.

*Runtime self-adaptation.* The ability to dynamically adapt to varying conditions, such as sensor availability and evolving clinical needs, is crucial for maintaining reliable patient care. This adaptability ensures continuity by providing consistent and accurate monitoring, even when sensors fail or become unavailable, through alternative data integration and software adjustments. Furthermore, it supports personalized treatment by dynamically modifying treatment plans and monitoring protocols in real time to align with the patient’s changing clinical needs. By optimizing tasks and parameters based on sensor availability and clinical needs, this capability improves resource utilization and healthcare efficiency in IoMT systems.

*Configurability.* In the field of IoMT, it is crucial for systems to be designed with the ability to be configured according to various aspects, including diverse health profiles and clinical needs of patients, user preferences, and available technological resources. This configurability at the point of instantiation allows for tailored solutions that can address unique healthcare protocols and individual needs effectively. By accommodating these diverse requirements, IoMT systems can improve their functionality, user experience, and overall performance in real-world medical environments.

Based on the importance of these non-functional aspects (i.e., interoperability, sustainability, security, runtime self-adaptation, and configurability) this article formulates five research questions, each focusing on one of these critical dimensions in IoMT systems.

To answer these research questions, we conducted a thorough review of the existing literature. The literature was identified and analyzed using the widely accepted methodology of a systematic literature review, as outlined by Kitchenham [2]. Following this rigorous process, a total of 280 articles were included and entirely examined. Many of these articles focus on specific aspect of IoMT systems (e.g., sensors integration, data exchange, data interpretation). However, it is noteworthy that a significant number are primarily concerned with the development of functional solutions, often neglecting or giving minimal attention to non-functional aspects. This highlights a gap in the current research, underscoring the need for a more comprehensive exploration of non-functional requirements within IoMT solutions.

The article is organized as follows. Section 2 provides an introduction to IoMT, presenting an overview of its key components. Section 3 presents a review of related work, highlighting existing surveys in the field. Section 4 outlines the methodology used for the analysis, including the research questions that guided our study. Section 5 presents the results of the analysis. Section 6 synthesizes the key findings and proposes future research directions, particularly focusing on addressing the non-functional requirements analyzed throughout the study. Finally, Section 7 discusses potential threats to the validity of the findings and concludes the article.

## 2. Internet of Medical Things

The term Internet of Things (IoT) refers to networks of computational devices that communicate via specific Internet protocols and technologies. The core idea is that these devices can connect and exchange data with each other and central systems autonomously, enabling real-time monitoring, environmental awareness, and intelligent responses to various events or conditions [3]. IoMT is a specific application of IoT in healthcare context. It refers to networks of medical devices, sensors, wearables, and other healthcare-related technologies that are interconnected through the Internet to collect, transmit, and analyze medical data. IoMT aims to improve medical processes by providing the means for real-time patient monitoring and timely and accurate diagnoses [4].

**Figure 1:**
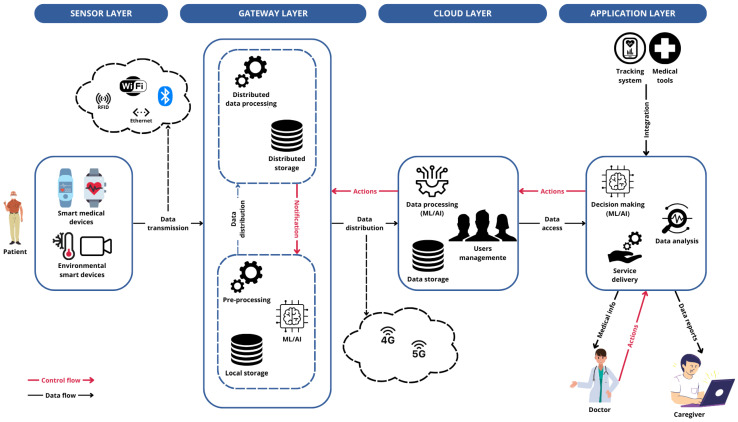
Common IoMT architecture consisting of four layers. For each layer, the main responsibilities are indicated. Highlighted are the data flow (black arrows) and the control flow (red arrows) between layers; particular communication technologies are also indicated.

Many IoMT systems adhere to a common architecture consisting of four distinct layers (see Figure 1): the *sensor* (or *perception*) layer, the *gateway* layer, the *cloud* layer, and the *visualization* (or *action*) layer.

Patients are equipped with sensors and devices capable of capturing a wide range of signals, including both physiological data and environmental factors. In the context of IoMT, these devices transmit the collected data via wireless or wired technologies to a gateway. The gateway is responsible for collecting the data and, if necessary, pre-processing them to optimize their use based on application needs. Through the gateway, data are sent to the cloud for storage and for more complex tasks that require greater computing resources. The cloud manages user data and makes them available at the application level for querying and further analysis. Authorized users can access the collected health data through the application interface, customize the collection parameters, or configure the system according to their specific needs. The cloud processes these configurations and forwards them to the appropriate gateway.

### 2.1. Sensor Layer

The sensor layer is the foundational layer of IoMT systems’ software architecture. It includes various data sources such as smart objects, monitoring devices, and mobile apps, each of which may utilize different types of sensors. These devices and sensors collect diverse signals, convert them into digital formats, and transmit the data using both wired and wireless communication technologies [1].

Actuators and controllers can be used to manage sensing devices and perform actions, such as opening and closing valves to control the flow of fluids or gases based on sensor readings [5]. Additionally, having a small amount of local storage can be useful for managing periods of lost connectivity with the gateway [1].

### 2.2. Gateway Layer

This layer is mainly responsible for data aggregation from the different data sources in the sensor layer, through short-range communication technologies [1]. This functionality is typically implemented in devices such as hubs or gateways located at the network’s periphery, close to the data acquisition devices (a concept often referred to as the *edge*). These devices perform relatively simple tasks such as data pre-processing, cleaning, and validation. They generally have limited storage capacity and use compact machine learning models for data processing [6]. In some cases, the gateway layer also includes the *fog* computing environment, a decentralized approach to computing and storage located closer to the data source [5]. The gateway layer is then responsible for distributing data to the cloud layer using long-range communication technologies [1].

### 2.3. Cloud Layer

In the cloud layer, high computational tasks on data are carried out, often utilizing AI and machine learning models for data processing [6]. Both centralized and distributed data storage are used for long-term data management. User management functions, such as registration, authentication, and access control, are implemented in the cloud [1]. Data analysis tasks can also be performed in the cloud [5].

### 2.4. Application Layer

The application layer is responsible for advanced data analysis tasks, leveraging machine learning and AI techniques to extract meaningful insights from collected data. This layer delivers application-specific services, such as data visualization, reporting, and decision support tools that enable healthcare professionals to adjust therapies and manage patient care more effectively [1]. Additionally, the application layer integrates external systems, including remote patient monitoring, tracking systems, and other medical devices, ensuring a cohesive and comprehensive healthcare management approach [5].

## 3. Related Work

In recent years, the healthcare sector has embraced advanced computing technologies to not only support providers but also enhance patient care. These innovations enable personalized treatment, real-time monitoring, and improved access to health data, offering patients more responsive and effective healthcare solutions. IoMT has gained significant attention, with several studies exploring its potential to connect medical devices and systems, enhancing the integration and coordination of healthcare services.

Several surveys have explored the evolving landscape of IoMT systems, often concentrating on technological and structural aspects, but with varying attention to security and privacy. Arbaoui et al. [7] examine state-of-the-art computing technologies for healthcare, focusing on the characteristics and benefits of edge and fog computing. They review various IoT architectures that integrate edge, fog, and cloud computing, proposing hybrid solutions for healthcare without deeply addressing security or privacy issues. Similarly, Huang et al. [8] provide an overview of IoMT from its conceptual foundations to practical deployment, highlighting the involved technologies and future directions but with limited emphasis on security concerns. Dwivedi et al. [1] conduct a systematic review of IoMT applications in healthcare, demonstrating their benefits for patients and providers while briefly mentioning challenges in developing smart healthcare systems, with minimal focus on data security and privacy protections.

In contrast, other surveys have placed a stronger emphasis on addressing security and privacy concerns within IoMT systems. Martínez et al. [9] contribute significantly to understanding security and privacy in healthcare by establishing a comprehensive foundation that includes the collection of specific security, privacy, and safety requirements for the medical domain. Their research classifies and maps threats using established frameworks and identifies existing security mechanisms and datasets relevant to AI applications, ensuring that healthcare systems are equipped to protect sensitive patient data from potential threats. Ashfaq et al. [10] also address security and privacy issues in IoMT systems, exploring the importance of reliable and secure data transmission and emphasizing the role of secure communication technologies alongside cloud-based and edge computing architectures. Their work highlights the critical need for robust security measures to ensure accurate and safe data handling in IoMT applications.

In reviewing the current literature on IoMT systems, it appears that existing surveys mainly focus on analyzing technologies and systems from a layered perspective, emphasizing their structural components. While these surveys provide valuable insights into the technological and architectural aspects, they often overlook crucial cross-cutting issues such as security, interoperability, runtime self-adaptation, and configurability.

Although some researchers have addressed security concerns, many surveys do not fully explore other essential aspects that can impact the robustness, versatility, and adaptability of IoMT solutions. The absence of comprehensive analysis on these dimensions creates a gap in understanding how to design systems that can dynamically adapt to varying conditions, ensure secure and seamless operation across different platforms, and maintain configurability to meet diverse and evolving healthcare needs.

Our survey aims to address this gap by providing a thorough analysis that delves deeply into interoperability, sustainability, security, runtime self-adaptation, and configurability. This work seeks to highlight the challenges and opportunities in designing adaptable, sustainable, secure, and interoperable healthcare solutions that can effectively respond to the complex and evolving demands of the medical field. By considering these critical aspects, our research contributes to a more complete understanding of the reliability and adaptability required for effective remote monitoring and overall system performance.

## 4. Method

The aim of our work is to investigate how proposals related to IoMT address open challenges, namely *interoperability*, *sustainability*, *security*, *runtime self-adaptation*, and *configurability*.

### 4.1. Research Questions

Our study is structured around five main research questions.

**RQ1: How do proposed solutions support interoperability between different digital healthcare systems?** This research question aims to explore the different strategies and technological solutions proposed in the literature to address interoperability challenges. Specifically, it seeks to identify and analyze how the proposed solutions facilitate the integration and communication among different digital healthcare systems, platforms, and devices.

For addressing this RQ, we investigate whether the selected articles report solutions that are compliant with a standard for the data format and the communication between systems. We also want to analyze if the proposed solutions integrate some third party services or expose their own services to external systems.

**RQ2: How is sustainability addressed in IoMT systems?** This research question explores how sustainability is addressed in IoMT systems, focusing on the techniques implemented for resource optimization.

Specifically, we examine whether the proposed solutions incorporate resource optimization techniques and identify which approaches are employed. By minimizing the resources required to perform tasks (e.g., through efficient algorithms, streamlined data processing, and reduced communication overhead), systems can operate more effectively, leading to significant energy savings. This dual focus not only enhances system performance but also aligns with sustainability goals by lowering the overall environmental impact of IoMT operations.

**RQ3: How is security ensured in IoMT systems?** This research question aims to evaluate the effectiveness of IoMT solutions in safeguarding sensitive medical data, preventing unauthorized access and ensuring the integrity and confidentiality of patient information.

To address this RQ, we conduct an analysis of the methodologies and techniques used for guaranteeing the confidentiality, integrity, and availability (CIA) triad. CIA triad is a set of guiding principles to ensure information security in IT systems [11]. In particular, confidentiality means that only authorized users can access to protected data. In IoMT context, it means that medical data have to be protected from unauthorized users during storing and transmitting. Integrity implies protecting data from unauthorized alteration (update, delete). In IoMT context, it means that medical data are not corrupted or deleted during storing and transmitting. Availability requires providing timing and uninterrupted access to data and services. In IoMT context, it means ensuring the continuous operation of medical devices and services. Special attention should be given to emergency services, which must respond promptly.

**RQ4: How do the proposed solutions support runtime self-adaptation according to sensors’ availability and clinical needs in IoMT systems?** This research question aims to explore how proposed solutions facilitate runtime adaptation within IoMT systems. By examining the proposed strategies and technologies, we seek to understand how these systems can adjust their operations in real-time to ensure continuous, personalized, and efficient healthcare delivery.

In particular, to address this RQ, we investigate how IoMT systems are designed to autonomously adjust and optimize their operations in response to dynamic conditions and changing requirements. This includes evaluating the implementation of self-adaptive mechanisms such as automated configuration adjustments (e.g., sensor availability or varying clinical needs of patients), real-time performance tuning, and responsive adjustments to varying operational environments, including unavailability and failure. By examining these aspects, we aim to understand how well IoMT solutions can maintain optimal functionality and reliability without requiring manual intervention, thus improving their resilience and adaptability in various healthcare settings.

**RQ5: How do the proposed solutions are customizable with respect to the available technological environments and the clinical needs?** This research question aims to investigate how IoMT systems are designed to be tailored to accommodate unique patient characteristics, integrate with available technological resources, and meet specific clinical requirements. By examining these aspects, we seek to understand the degree of customization and personalization offered by IoMT solutions, ensuring they can effectively address the diverse needs of healthcare environments.

To address this research question, we analyze the configuration processes described in any of the proposed solutions. This includes evaluating the ability of the solutions to customize settings based on patient-specific data, available technologies (sensors and software modules), and clinical needs.

### 4.2. Systematic Review

We conducted a systematic review of the peer-reviewed literature to capture the latest advances and proposals in the Internet of Medical Things (IoMT) domain. The review process was designed to answer the five research questions defined in this study, with a particular focus on non-functional requirements such as interoperability, sustainability, security, runtime self-adaptation, and configurability.

**Research strategy**. To identify relevant studies, we performed a comprehensive search across multiple electronic databases: the ACM Digital Library, IEEE Xplore, Scopus, PubMed, and Google Scholar.

For each database, we used the search query string (“Internet of Medical Things” OR “IoMT” OR “Internet of Healthcare Things” OR “IoHT” OR “smart health” OR “ehealth” OR “telehealth” OR “mhealth”) AND (“Digital Twin” OR “Point of Care” OR “PoC” OR "Edge” OR “Integration” OR “Heterogenous”). These keywords were chosen to ensure comprehensive coverage of the key aspects related to interoperability, sustainability, security, self-adaptation, and customization in IoMT systems.

The first set of keywords (i.e., “Internet of Medical Things” and “IoMT”, “Internet of Healthcare Things” and “IoHT”, “smart health”, “ehealth”, “telehealth”, and “mhealth”) captures the broad domain of digital healthcare and IoMT-related technologies. These terms encompass various healthcare solutions, from connected medical devices to telemedicine and mobile health applications, ensuring that relevant studies across different healthcare paradigms are included.

To refine the search towards studies that specifically address our research questions, we included additional terms relevant to specific technical and functional aspects of IoMT systems. “Digital Twin” was included to cover approaches that enhance interoperability and system adaptability through virtual representations of physical healthcare environments (RQ1, RQ4). “Point of Care” and “PoC” were added to capture studies focusing on real-time and decentralized healthcare solutions, which are crucial for addressing sustainability (RQ2). Furthermore, “Edge” was included to identify architectures that enhance security, reduce latency, and optimize resource usage in IoMT systems, thus contributing to the discussion on security, sustainability, and customization (RQ2, RQ3, RQ5). Finally, “Integration” and “Heterogeneous” were selected to capture research focused on interoperability and the ability to customize IoMT solutions to different technological and clinical environments (RQ1, RQ5).

This combination of keywords ensures that the retrieved literature is relevant to the core topics of our survey while maintaining a broad enough scope to include diverse technological approaches and application domains within IoMT. Although our analysis focuses on non-functional requirements, the selected keywords were intentionally broader and more inclusive. This strategy was adopted to capture a wide spectrum of IoMT-related solutions that may address these aspects implicitly, even when not explicitly mentioned in the title or abstract.

For each database, we extracted the first 200 results ranked by relevance, for a total of 1000 articles. To ensure the inclusion of the most recent advancements and trends in the field, we limited the selection to works published between January 2020 and January 2024.

**Inclusion criteria.** To be included in the review, studies had to meet the following criteria:

- Be written in English and published in a peer-reviewed venue.
- Present a concrete solution (e.g., method, technique, model, or architecture) aimed at addressing at least one of the five non-functional requirements under investigation.
- Provide sufficient technical depth, such as implementation details, validation strategies, or architectural frameworks.
- Clearly fall within the domain of the Internet of Medical Things (IoMT), with relevance to medical devices, healthcare infrastructure, or clinical data.

These criteria ensured that the selected literature would be both recent and directly relevant to the non-functional dimensions under analysis, while excluding general-purpose IoT or conceptual papers lacking technical contributions.

**Screening and eligibility.** After removing 218 duplicates, 782 unique articles remained. We conducted an initial screening based on abstracts, introductions, and conclusions to exclude papers that were clearly unrelated to the scope of our study. This led to the exclusion of 502 papers, resulting in 280 articles considered for full-text eligibility assessment.

Each of the 280 full-text articles was independently analyzed to assess its relevance with respect to the five non-functional requirements. We focused on the presence of explicit techniques, architectural proposals, or evaluations targeting one or more of these aspects. To ensure consistency, we applied the following guiding questions during the inclusion process:

- Does the article introduce or evaluate a method/architecture addressing at least one of the five non-functional requirements?
- Is the discussion of the non-functional requirement supported by technical depth (e.g., implementation, modeling, validation)?
- Is the context of application clearly situated within IoMT (i.e., healthcare-specific, device-connected infrastructure)?

While no formal scoring system was applied, the relevance assessment was guided by shared inclusion criteria agreed upon in advance. Articles were reviewed consistently, and when minor uncertainties emerged (e.g., borderline relevance to a non-functional requirement), the decision was made collaboratively to ensure alignment with the study scope.

**Exclusion criteria.** A total of 204 articles were excluded at this stage, based on the following:

- **Out-of-scope focus**: The paper addressed functional aspects (e.g., sensing modalities or clinical applications) with no meaningful discussion of non-functional requirements.
- **Insufficient technical depth**: Mentioned non-functional aspects only superficially, with no methodological or architectural contributions.
- **Lack of concrete solution**: No specific technique, model, or method addressing the requirements was proposed.
- **Non-IoMT context**: The system addressed was not specific to the medical domain.
- **Redundancy**: Duplicate content across conference/journal versions; only the more complete version was retained.

As a result, 75 articles were included in the final analysis.

**Figure 2:**
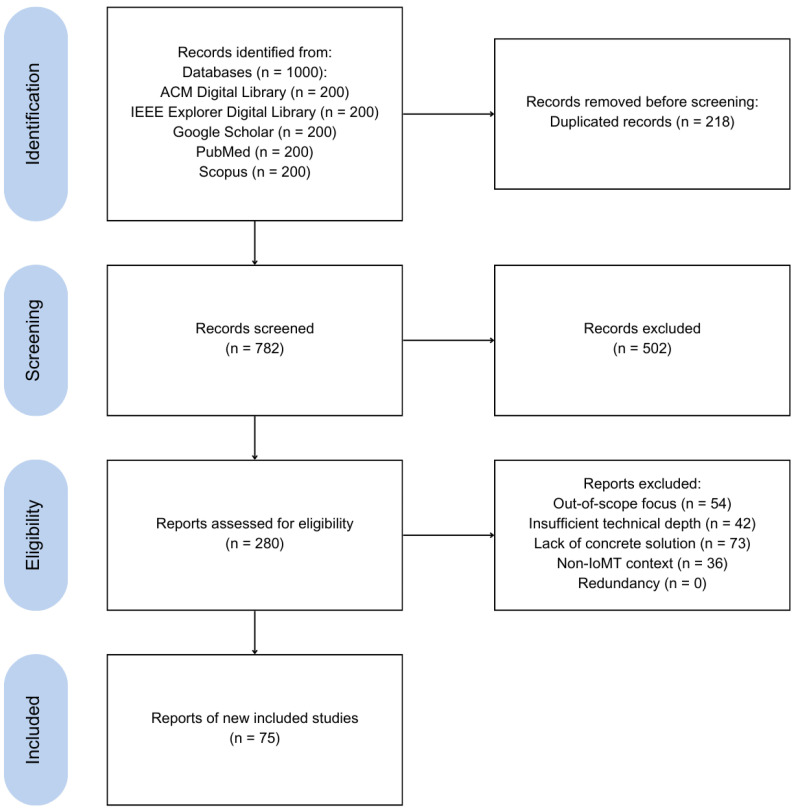
Application of the PRISMA methodology in our survey, highlighting the number of articles selected at each step of the literature selection, analysis, and synthesis process.

Figure 2 provides an overview of the systematic review process, following the PRISMA (Preferred Reporting Items for Systematic Reviews and Meta-Analyses) guidelines [12]. It illustrates the flow from initial identification and screening through to eligibility and inclusion, highlighting the number of studies retained or discarded at each step.

**Figure 3:**
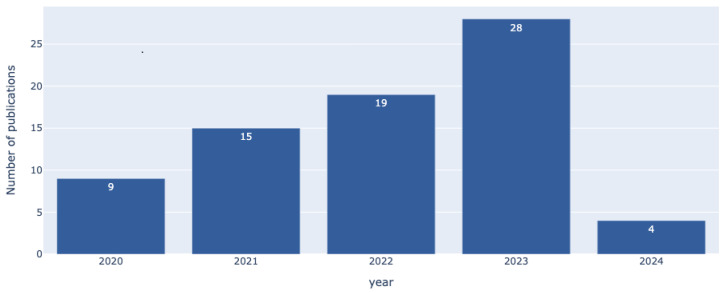
Distribution of selected articles by year of publication (2020–2024).

Figure 3 shows the temporal distribution of the selected studies. As illustrated, the number of relevant publications has steadily increased year by year, reflecting growing research interest in the non-functional dimensions of IoMT systems.

## 5. Results

The section is organized into subsections, each one addressing a specific research question from the five we formulated.

### 5.1. RQ1: Interoperability

The solutions for achieving interoperability generally focus on three main issues: *(i) standards for data formatting*, *(ii) integration of third-party systems’ services*, and *(iii) exposure to third-party systems’ services*.

#### 5.1.1. Standards for Data Formatting

In terms of *standards for data formatting*, several approaches have been adopted. One of the most notable is Fast Healthcare Interoperability Resources (FHIR) by Health Level 7 (HL7) (https://hl7.org/fhir/), which provides a robust framework for data formatting and interoperability across healthcare systems. In this context, Tsiouris et al. [13] adopt the FHIR data model for their proposed system. By using FHIR, their approach aligns with a widely accepted standard, ensuring that data can be effectively exchanged and stored across different platforms.

Another significant standard is OpenEHR (https://openehr.org/), which Rubí et al. [14] employ in their system. OpenEHR is used to structure healthcare data, and Rubí et al. enhance its functionality by integrating it with Semantic Sensor Network ontologies (https://www.w3.org/TR/vocab-ssn/, accessed on 31 May 2024). This combination not only adheres to the OpenEHR standard, but also aims to achieve semantic-level interoperability, enabling a deeper and more meaningful exchange of data.

Additionally, Bui et al. [15] define their own data model but incorporate established standards such as Open mHealth (https://www.openmhealth.org/) where applicable. Their approach benefits from existing frameworks while customizing the model to address specific needs, thus providing a balance between leveraging established standards and meeting particular system requirements.

#### 5.1.2. Integration of Third-Party Systems’ Services

Among the solutions for the *integration of third-party systems’ services*, several approaches are identified. One such approach is presented in the system proposed by Bui et al. [15], where the server is designed to connect with smart sensor platforms that do not utilize Bluetooth Low Energy (BLE) communication. This integration is achieved through a specific platform API, which enables the server to retrieve the collected data from these sensors. In contrast, the system proposed by Tsiouris et al. [13] integrates Fitbit trackers using the Fitbit API and supports various other devices and sensors. Additionally, it enables communication with other user devices, healthcare services, and registries by leveraging the FIWARE-NGSI v2 (a RESTful API for managing context information in smart systems, enabling standardized data exchange across heterogeneous IoT applications), thanks to the integration of the FIWARE-Orion message broker (a context broker that implements the NGSI-v2 standard, enabling publish/subscribe interactions for managing real-time context information in IoT applications).

#### 5.1.3. Exposure to Third-Party Systems’ Services

Among the solutions for *exposure to third-party systems’ services*, two main approaches have been identified. One approach leverages blockchain technology to ensure secure data access. For instance, Fourati et al. [16] utilize a public blockchain to provide data and analytics access to external entities, such as scientific communities and public health authorities. Similarly, Hewa et al. [17] employ blockchain by providing an API that grants trusted third parties access to patient data, which requires obtaining patient consent and ensuring data anonymity. Rachakonda et al. [18] adopt a similar strategy, storing collected data on a blockchain and providing secure API access to third parties, while maintaining user privacy. The second approach focuses on integrating with existing healthcare services through standardized APIs. Tsiouris et al. [13] implement an FHIR-compliant REST API to facilitate interaction with healthcare services, ensuring adherence to established standards for data exchange. Rubí et al. [14] use a REST API to expose OpenEHR data. Their system serves two purposes: it provides OpenEHR data to legacy hospital applications and extends OpenEHR-OWL Ontology data to new semantic applications, highlighting the versatility of their approach in integrating both traditional and emerging systems.

Table 1 provides an overview of the articles analyzed for RQ1. The table organizes the articles into categories based on standards for data formatting, integration with third-party systems, exposure to third-party systems, and the use of standards or API definitions.

### 5.2. RQ2: Sustainability

The sustainability solutions found in the reviewed articles are often related to managing specific Quality of Service (QoS) parameters. Among these, delay and latency are commonly addressed. Delay refers to the total time taken between the sending and receiving nodes and includes communication, propagation, data processing, and queuing delays [19]. Latency, in contrast, is defined as the sum of propagation and processing time between the sending and receiving nodes, excluding queuing and buffering delays [20,21]. Additional parameters that influence sustainability include processing time, CPU usage, network traffic, and storage capacity.

While not all solutions explicitly justify their optimizations as efforts to reduce energy consumption or minimize environmental impact, these resource-efficient practices inherently contribute to greener computing by promoting lower energy use and enhancing overall system efficiency.

The main techniques adopted to ensure sustainability in IoMT systems can be categorized into four key areas. First, *distribution algorithms* are employed to optimize system resources by distributing computational tasks according to specific criteria. This includes scheduling, task offloading, and load balancing strategies. Second, *event-based algorithms* are utilized, implementing strategies that trigger actions based on specific events, which helps in dynamically managing system operations. Third, *optimization algorithms*, such as heuristics, machine learning models, and other targeted techniques, are designed to enhance system efficiency and resource utilization. Lastly, *distributed computing* plays a crucial role by introducing intermediate architectural layers, such as fog and mist computing (mist computing involves data processing closer to the data source, using lightweight devices such as sensors and microcontrollers), between edge devices and the cloud, which helps to reduce latency and improve energy efficiency by processing data closer to the source.

#### 5.2.1. Distribution Algorithms

Among the solutions implementing *distribution algorithms*, several approaches focus on reducing latency, optimizing resource use, and enhancing energy efficiency. Alatoun et al. [20] develop a task offloading strategy that assigns tasks to the nearest fog node based on task priority, helping to reduce latency and network usage while improving energy efficiency by minimizing CPU usage on fog computing nodes. Similarly, Almudayni et al. [22] introduce a mist broker that offloads tasks considering the patient’s health condition and the computational capacity of available nodes across mist, fog, and cloud layers. This strategic distribution reduces processing time and latency, ultimately leading to lower energy consumption. Rajpoot et al. [23] utilize an Ant Colony Optimization-based load balancing technique to decrease response time and minimize delays, emphasizing efficient resource utilization. Yadav et al. [21] propose a task offloading scheme that uses reinforcement learning to identify optimal resource nodes, balancing energy consumption and latency based on the current state and actions to be performed by computing nodes.

#### 5.2.2. Optimization Algorithms

Among the solutions implementing *optimization algorithms*, several approaches share similar goals of enhancing system efficiency and reducing resource consumption. Idrees et al. [24] introduce a lossless data compression technique that reduces latency, storage, and network usage, contributing to energy efficiency during data processing and transmission. Jazaeri et al. [25] propose a Moth-Flame Optimization caching algorithm that optimizes data access, thereby minimizing latency. Rajpoot et al. [23] utilize the Ant Colony Optimization method for load balancing, effectively reducing delays. Yadav et al. [21] employ reinforcement learning for task offloading, dynamically identifying optimal resource nodes to balance energy consumption and latency. Similarly, Gharaei et al. [26] develop an optimization algorithm that manages wireless chargers to enhance energy efficiency by minimizing charging time.

#### 5.2.3. Distributed Computing

Among the solutions utilizing *distributed computing*, several approaches strategically position computing resources closer to the data source to optimize performance and reduce resource consumption. Alatoun et al. [20] propose a fog environment, positioning schedulers and computing nodes near the edge to address network usage, latency, and the energy constraints of edge devices. This proximity minimizes the distance between devices and computing nodes, resulting in reduced energy consumption. Almudayni et al. [22] introduce a mist layer that optimizes task allocation, effectively managing processing time and latency while strategically offloading tasks to lower energy consumption. Similarly, Mukherjee et al. [19] employ fog nodes alongside cloud resources, using fog devices as health evaluation nodes to decrease delay and reduce the energy usage of user devices, demonstrating efficiency compared to purely cloud-based approaches. Rajpoot et al. [23] also leverage fog computing, transferring computational tasks to the fog environment to minimize delays, showcasing the effectiveness of distributed computing in enhancing the sustainability and performance of IoMT systems.

#### 5.2.4. Event-Based Algorithms

Among the solutions employing *event-based algorithms*, various approaches are designed to optimize resource usage by triggering actions only under specific conditions, thereby conserving energy and computational resources. Gharaei et al. [26] propose an approach where a health index representing the patient’s condition is updated and stored only when changes occur. This strategy reduces the amount of stored data, network usage, and cloud CPU utilization. Similarly, Mukherjee et al. [19] propose a system where high-computation tasks are offloaded to the cloud only if the patient’s health condition appears not within normal parameters, as detected by fog nodes. This selective offloading minimizes energy consumption by avoiding unnecessary data processing. Emish et al. [27] implement a solution where GPS sensors are activated only when movement is detected, relying on other motion sensors for position data at other times, which significantly lowers the energy usage of wearable devices. In a related approach, Saini et al. [28] introduce event-based data transmission, where data is sent only when an event of interest is detected, thus conserving energy in devices with limited power resources.

#### 5.2.5. Lifecycle and Trade-Offs in Practice

Real-world deployments demonstrate the practical benefits and challenges of sustainable IoMT architectures. In hospital settings, fog nodes enable local processing and reduce cloud reliance [19,22], lowering energy use and improving responsiveness. However, offloading tasks to edge devices introduces trade-offs: while latency and network traffic are reduced, gateway nodes face higher processing loads [20,21].

Several of the reviewed techniques (e.g., task offloading, opportunistic data transmission, and event-driven architectures) contribute to sustainability by limiting unnecessary data flow and optimizing resource utilization [20,26,27]. When combined with lightweight protocols (e.g., CoAP, 6LoWPAN) and context-aware scheduling, these methods support greener computing [21,24].

Beyond runtime strategies, recent studies advocate addressing the full lifecycle of IoMT devices. Predictive maintenance and modular hardware design can extend device lifespan and facilitate reuse [29,30]. However, broader environmental indicators (e.g., recyclability, emissions, and end-of-life management) are still rarely addressed in IoMT evaluations [31]. This underscores the need for more comprehensive sustainability metrics.

Table 2 summarizes the results related to RQ2, highlighting the dimensions addressed by the examined approaches, such as latency, energy consumption, and resource usage. The rows list these dimensions, while the columns show the techniques used, including distribution algorithms, optimization algorithms, distributed computing, and event-based algorithms.

### 5.3. RQ3: Security

To address RQ3, the analysis of security mechanisms in IoMT systems is structured into two distinct sections, focusing on how various technologies ensure the principles of confidentiality, integrity, and availability (CIA). This structure allows for a comprehensive evaluation of the diverse strategies adopted in the literature to protect sensitive medical data, prevent unauthorized access, and ensure system resilience.

Section 5.3.1 examines technologies and methodologies designed to guarantee confidentiality and integrity. These aspects are often addressed through *authentication*, *access control*, and *encryption* mechanisms. Both blockchain-based and non-blockchain solutions are explored, highlighting their role in restricting access to authorized users and ensuring data remain unaltered. In blockchain contexts, cryptographic techniques such as encryption and hashing inherently support these objectives, while smart contracts automate data governance and access management.

Section 5.3.2 focuses on ensuring availability, which is critical for the continuous operation of IoMT systems. The discussion addresses technologies for both *anomaly detection* and *intrusion detection*, which are essential for identifying and mitigating threats that could disrupt system functionality. Blockchain’s decentralized architecture naturally enhances availability through redundancy and fault tolerance, while non-blockchain solutions achieve similar outcomes using diverse monitoring and detection strategies.

#### 5.3.1. Confidentiality and Integrity

Ensuring confidentiality and integrity of data within IoMT systems necessitates a combination of robust authentication mechanisms, effective access control policies, and encryption techniques. Each of these dimensions employs specific technologies and approaches to secure data and prevent unauthorized access.

Table 3 presents the technologies supporting confidentiality and integrity, categorized by feature (authentication, access control, and encryption) and further subdivided by approach.

##### Authentication

Authentication is fundamental for verifying the identity of actors interacting with an IoMT system, thereby safeguarding sensitive medical data. The reviewed literature reveals a variety of authentication techniques categorized by the underlying technology and approach.

**Digital signatures** are widely used for ensuring data authentication. One of the most common forms of digital signature used in IoMT systems is the elliptic curve-based digital signature (ECDS), which provides strong security with relatively small key sizes, making it ideal for resource-constrained devices in IoMT environments. For instance, Sriborrirux et al. [38] propose mutual authentication between devices, gateways, and the cloud platform in a microservice-based IoMT framework using ECDS.

Several studies integrate blockchain technology to enhance the security and efficiency of authentication in IoMT systems, leveraging its decentralized nature to store identity data and automate authentication through smart contracts [16,32,33,34,35,36,37]. For example, Khan et al. [34] employ a federated learning framework where local nodes are authenticated with ECDS, ensuring secure real-time data sharing for patient monitoring. Similarly, Egala et al. [37] use ECDS to authenticate devices in a decentralized healthcare cyber–physical system. Some solutions adopt multi-level blockchain architectures, combining global and local blockchains to address diverse authentication needs. For example, Alsaeed et al. [33] propose a lightweight group authentication framework where a global blockchain acts as a decentralized identity registry, while local blockchains handle device authentication in fog areas. Akkaoui et al. [36] present a framework where local blockchains authenticate users and devices through ECDS, and a global blockchain secures off-chain medical record hashes, ensuring both identity management and data integrity. Other approaches incorporate multi-step authentication processes. For instance, Vangipuram et al. [35] implement a multi-factor system combining credentials and one-time passwords (OTP), with ECDS automated through smart contracts to authenticate messages, adding an extra security layer to blockchain-assisted IoMT environments. A different approach to digital signature technology is the use of attribute-based digital signatures (ABDS), which enable more fine-grained access control by allowing authentication based on the attributes or roles of users rather than their identity alone [39,40]. For instance, Guo et al. [39] propose a hybrid blockchain-edge architecture for managing and sharing electronic health records (EHR), in which users are authenticated using ABDS.

Another approach involves using asymmetric digital signatures, relying on public–private key pairs to ensure security through data encryption and signature verification, thus maintaining the integrity and authenticity of messages within IoMT systems [18]. In some frameworks, digital signatures are used without specifying the type, focusing on their role in automating authentication and ensuring data integrity. For example, Alruwaill et al. [41] propose a blockchain-based smart healthcare system for continuous patient monitoring with IoMT devices. The system implements a two-step authentication process: user authentication based on credentials and GPS location, followed by data authentication via digital signatures, all automated through smart contracts.

**Digital signature-based certificates** strengthen authentication by linking public keys to their owners through digital signatures, verifying entity identities and ensuring data integrity. Some approaches rely on elliptic curve signature certificates. For example, Kumar et al. [42] propose a system that secures acquired data using blockchain and the interplanetary file system (IPFS) (https://ipfs.tech/), where users are authenticated through these certificates. Other solutions use elliptic curve Qu-Vanstone certificates, such as the one proposed by Hewa et al. [17] in a multi-access edge computing and blockchain-based service architecture. Finally, some solutions do not specify the certificate type. For instance, Zaabar et al. [43] present a blockchain-based system for secure and privacy-aware remote patient monitoring, where users are authenticated using digital signature-based certificates.

**Identity-based** authentication uses credentials tied to a user’s identity (e.g., usernames, passwords, digital certificates) to verify access, directly linking the authenticated identity to authorized resources. In IoMT systems, there are two main approaches: traditional credential-based methods [13,25,49,50,51] and those incorporating smart contracts [44,45,46,47,48]. The first approach relies on centralized authentication, where user credentials are stored and verified by a server or device. Various frameworks use service nodes or cloud providers to manage user profiles and authenticate users through credentials. For example, Ramzan et al. [49] propose a system where service nodes authenticate users through credentials, while Jazaeri et al. [25] enable device authentication for health data sharing. Tsiouris et al. [13] describe a platform using server-side authentication with credentials and JSON Web Tokens (JWTs), and Bergoeing et al. [50] incorporate credentials for user authentication in their AI-integrated mobile health platform. Similarly, Panja et al. [51] design an IoMT framework for monitoring COVID-19 patients, relying on cloud providers for user authentication. The second approach integrates smart contracts into the authentication process, leveraging blockchain technology for user verification. For example, Khan et al. [45] utilize patient identities stored on the blockchain for authentication in a healthcare data-sharing system, while Zaabar et al. [46] introduce an intrusion detection system for wearable health devices, authenticating users or nodes via blockchain-stored identities. Makina et al. [47] combine edge computing, blockchain, and IPFS with user authentication using blockchain-stored credentials. Additionally, Hennebelle et al. [48] propose an IoT–edge–AI–blockchain system for diabetes prediction, where authentication is based on user IDs and secret PINs.

Advanced schemes, such as **multi-factor** authentication, provide an additional layer of security by combining multiple authentication factors [35,41,52,53].

Other methods include the use of **physical unclonable functions** (PUF), which ensure hardware-level identity security [32,54], and the use of **authentication schema**, which enable privacy-preserving verification [48,55,56,57,58].

##### Access Control

Access control mechanisms in IoMT regulate who can access, modify, or interact with resources, ensuring that only authorized users, devices, or entities can perform specific actions. These mechanisms protect sensitive data and medical equipment by enforcing permissions based on factors such as user roles or device types. In IoMT, they are crucial for safeguarding patient privacy, ensuring system security, and meeting regulatory requirements.

**Digital signatures** are employed in several solutions to validate access control policies, particularly those leveraging elliptic curve cryptography (ECC). For example, Wazid et al. [58], Fourati et al. [16], and Vangipuram et al. [35] employ ECC-based digital signatures in blockchain-enabled systems to automate access control in healthcare environments. Attribute-based digital signatures are also commonly used to secure access to health data stored on the blockchain, as demonstrated by Guo et al. [39] and Ali et al. [40].

**Identity-based** mechanisms focus on establishing access rights based on the identity relationships between users and data owners. For example, Ramzan et al. [49] propose a decentralized framework where access control is enforced by service nodes verifying these identity relationships, while Khan et al. [45] and Hewa et al. [17] use smart contracts to store and validate such relationships on the blockchain, ensuring that only authorized users can access sensitive data.

**Role-based access control** (RBAC) is another prevalent method, where users are assigned roles that determine their access rights. In this context, Alsaeed et al. [33] and Bergoeing et al. [50] implement RBAC in their IoMT systems, with access restricted according to the roles assigned to each identity. These approaches do not rely on smart contracts for automation. On the other hand, several approaches exploit smart contracts to perform role-based access control. For instance, Akkaoui et al. [36] and Alruwaill et al. [41] automate role-based access using smart contracts coded into the blockchain, while Bang et al. [60] focus on controlling access based on both the role of the requester and the privacy conditions of the patient.

Other advanced approaches include **rule-based** systems, which enforce conditional policies [43,46], and innovative methods such as selective-ring-based [37] or triple-subject purpose-based access control systems, which provide enhanced flexibility in policy definition [61]. Additional features, such as access logging mechanisms [62] and approval-based multi-secret sharing systems, further enhance traceability and control in access management [51].

##### Encryption

Encryption is a crucial mechanism for safeguarding sensitive data, such as medical information, by converting it into a coded format accessible only to authorized parties. It ensures confidentiality by preventing unauthorized access, securing data during both storage and transmission.

**Symmetric encryption**, which uses the same key for both encryption and decryption, is widely employed in IoMT systems to secure data both at the edge and during transmission due to its computational efficiency. Algorithms such as lightweight encryption algorithm (LEA) are used to encrypt raw data collected from IoMT devices, as demonstrated by Bajpai et al. [63]. Similarly, data are often encrypted before being stored on blockchain platforms, as seen in the solutions provided by Akkaoui et al. [36] and Alruwaill et al. [41], who use symmetric encryption to ensure that health records remain secure and accessible only to authorized users. Furthermore, symmetric encryption is frequently used in scenarios involving the sharing of encrypted data among various stakeholders, as highlighted by Soleymani et al. [56] and Hewa et al. [17].

**Asymmetric encryption** techniques have been proposed alongside symmetric methods to ensure the security of sensitive medical data, with a particular focus on preserving patient privacy, securing transmissions, and safeguarding stored information. Homomorphic encryption, a method that enables computations to be performed on encrypted data without requiring decryption, has proven especially valuable in this context. Ali et al. [40] and Khan et al. [34] employ homomorphic encryption to secure electronic health records at the user side and during transmission, ensuring that sensitive information remains protected even in distributed environments. Similarly, Fourati et al. [16] utilize this technique to safeguard patient privacy, while Dang et al. [62] leverage it to protect model updates stored within blockchain infrastructures.

Homomorphic encryption is also applied in specific scenarios involving data aggregation and storage. Hakak et al. [64] adopt this approach to secure the sharing of local model weights with an aggregator server, and Bajpai et al. [63] employ Paillier encryption, a variant of homomorphic encryption, to securely aggregate data at the sink node. Guo et al. [39] extend this methodology to off-chain storage of electronic health records using the IPFS. Moreover, Salim et al. [65] apply homomorphic encryption to protect medical plain-text data, while Meng et al. [66] combine it with machine learning algorithms, such as XGBoost, to secure medical datasets during analytical processes. In addition to homomorphic encryption, RSA, a widely recognized asymmetric encryption technique, has been used for encrypting medical data stored on-chain. For instance, Rachakonda et al. [18] adopt RSA to ensure the security and integrity of medical information within blockchain systems. These diverse implementations highlight the adaptability and effectiveness of asymmetric encryption in meeting the complex security requirements of IoMT systems.

Elliptic curve cryptography (ECC) has emerged as a prominent asymmetric encryption technique due to its efficiency and strong security features. A number of studies have applied ECC to secure both data storage and communication. For instance, Wazid et al. [58], Nguyen et al. [44], and Vanjpuram et al. [35] utilize ECC to encrypt on-chain data, ensuring that sensitive medical information stored on blockchain platforms is well-protected. Similarly, Kumar et al. [42] and Egala et al. [37] apply ECC to encrypt both messages and data stored on-chain, securing communication and data integrity across distributed systems. In addition to these implementations for on-chain data, ECC has also been used for encrypting communication in IoMT systems. Alsaeed et al. [33] and Kumar et al. [67] rely on ECC to encrypt all messages exchanged between devices and servers, ensuring secure and private communication across the network.

Other asymmetric encryption techniques have also been explored in IoMT systems. For example, Ghanbarafjeh et al. [68] introduce a new attribute-based encryption algorithm, which dynamically encrypts patient data and messages, offering an additional layer of security by incorporating user attributes into the encryption process. Meanwhile, Makina et al. [47] employ an unspecified asymmetric encryption algorithm for off-chain data encryption, further illustrating the versatility of asymmetric techniques in securing medical data across various storage and communication environments.

Beyond common encryption techniques, other approaches have been explored for securing sensitive data in IoMT systems, each offering unique advantages based on the use case and system requirements. Secure Mmulti-party computation (SMPC) has been leveraged for preserving privacy during transactions in blockchain environments. Fourati et al. [16] apply SMPC to safeguard data while processing transactions on the blockchain, ensuring that sensitive information remains confidential even when multiple parties are involved. Similarly, Kalapaaking et al. [69] employ SMPC-based encryption in a federated learning framework, securing models while enabling collaborative learning without compromising data privacy. Another innovative technique is identity-based encryption, as demonstrated by Aggarwal et al. [57], who combine face biometry with a fuzzy identity-based encryption scheme to secure patient vital signs. This approach enhances security by linking encryption to biometric features, providing a robust mechanism for identity verification and data protection. Wei et al. [70] introduce a compressive encryption process paired with a deep learning-based sparse recovery algorithm. This sparse learning-based encryption scheme provides an efficient approach to data encryption, safeguarding privacy while enabling data recovery with minimal storage overhead. Liu et al. [55] explore certificateless proxy re-encryption, a mechanism used to secure model updates in federated learning. By encrypting noise vectors, this technique protects the transmission of sensitive information between participants and the aggregator server, enhancing both security and scalability in distributed learning environments. For securing data transmission, Emish et al. [27] and Jameel et al. [71] utilize the SSL protocol, which ensures encrypted communication between users’ browsers and web servers, as well as between gateways and servers, respectively.

#### 5.3.2. Anomaly & Intrusion Detection

To ensure availability, intrusion and attack detection systems are designed to promptly identify potential threats and implement appropriate countermeasures. Additionally, some systems integrate anomaly detection to recognize not only unusual behaviors indicative of malicious activity but also potential system faults.

In the context of the CIA triad, availability is considered a core security requirement in IoMT environments, particularly due to the critical nature of medical services that rely on timely and uninterrupted data exchange. Therefore, strategies that enhance availability are not limited to system reconfiguration or self-adaptation (as discussed in Section 5.4), but are also embedded within security mechanisms. For instance, many of the approaches analyzed in this section adopt distributed architectures, edge-level analytics, and federated learning to mitigate the risk of centralized failure and to preserve service continuity under adverse or dynamic conditions. These techniques contribute to system resilience by enabling fault detection, local autonomy, and quick recovery from attacks or malfunctions.

The techniques identified have been grouped into three categories: *attack detection*, *intrusion detection*, and *anomaly detection*. Attack detection focuses on identifying and mitigating specific malicious actions targeting the system, such as denial-of-service attacks or data breaches. Intrusion detection involves monitoring and analyzing network or system activity to uncover unauthorized access or policy violations. Anomaly detection, on the other hand, is designed to identify deviations from expected behavior, which may indicate either malicious activity or potential system faults.

Table 4 summarizes the techniques for attack detection, intrusion detection, and anomaly detection in IoMT systems, highlighting methods that use different computational models and learning paradigms.

##### Attack Detection

Attack detection predominantly employs machine learning techniques, with a focus on approaches such as incremental classifiers implemented at the fog layer to effectively identify potential threats. For example, Hameed et al. [72] propose a fog-computing architecture for IoMT systems, featuring a hybrid, lightweight multi-attack detection system that uses adaptive learning techniques to analyze network statistics and packet data for early and efficient attack detection.

##### Intrusion Detection

Intrusion detection is characterized by a diverse range of techniques spanning deep learning, machine learning, and federated learning. In the domain of deep learning, Al-Shammari et al. [73] present a framework for securing IoMT devices using a deep neural network (DNN) at the edge to analyze device operations. Zubair et al. [74] expand on this by employing a DNN classifier for detecting abnormal activities from BLE packets, while Aggarwal et al. [57] deploy a DNN-based system in the cloud to identify inter-domain malicious data streams. Faruqui et al. [75] propose a combination of convolutional neural networks (CNNs) and long short-serm memory (LSTM) networks to defend against intrusions by processing both sequential and grid data.

Federated learning approaches are increasingly being adopted for intrusion detection. For example, Singh et al. [76] integrate hierarchical LSTM models within a federated learning framework to reduce latency and improve accuracy. Khan et al. [77] employ reinforcement learning in a privacy-preserving federated learning model to detect cyber-attacks, while Zaabar et al. [46] introduce a blockchain-enhanced federated learning architecture that utilizes support vector machines (SVM) and random forest classifiers for detecting abnormal traffic in the network of wearable health devices. Additionally, Aljuhani et al. [78] propose a collaborative intrusion detection system framework that leverages fog computing for a more cost-effective and efficient detection mechanism.

##### Anomaly Detection

Anomaly detection in IoMT systems is typically addressed through machine learning, probabilistic models, and stochastic models. Machine learning techniques are commonly used, as seen in the work of Said et al. [79], where an SVM-based system is implemented to detect anomalies in patient health, environment, and network data in a smart hospital setting. Similarly, Zachos et al. [80] present a hybrid anomaly-based intrusion detection system that integrates operational data from IoMT devices and network traffic to identify potential issues.

In the context of probabilistic models, Khan et al. [34] propose a lightweight anomaly detection mechanism based on a Gaussian mixture model (GMM) for real-time identification of threats in IoMT systems, leveraging device operation data.

Stochastic models are also employed, as demonstrated by Gupta et al. [81], who developed an anomaly detection model for remote patient monitoring based on a hidden Markov model. This model is designed to detect anomalies in both device data and user behavior, addressing potential issues arising from malicious devices or critical user conditions, with the data being analyzed in the cloud.

### 5.4. RQ4: Self-Adaptation

In IoMT systems, the ability to self-adapt is essential for maintaining operational continuity and ensuring correct functionality in the face of dynamically changing environments. Indeed, these systems must respond to variations in device availability, network conditions, and patient health status, thus requiring mechanisms that enable real-time adjustments.

To address the research question on runtime self-adaptation in IoMT systems, it is important to distinguish between two main forms of adaptability: *technological adaptability* and *adaptability to patient conditions*. This distinction highlights the dual challenge IoMT systems face: responding to technical limitations while concurrently adjusting to the changing health conditions and treatment protocols of individual patients.

#### 5.4.1. Technological Runtime Adaptability

Technological runtime adaptability in IoMT systems is guided by the necessity to sustain operational efficiency in the context of dynamic and evolving technological environments. In the literature, this type of adaptability can be categorized based on specific goals: *sensor and data adaptability*, *failure handling*, and *resource optimization*.

##### Sensor and Data Adaptability

Sensor and data adaptability refers to the system’s capacity to dynamically adjust its behavior at runtime in response to the availability of sensors and data streams. A noteworthy approach comes from Hameed et al. [72], who propose a fog-based attack detection system that adapts to new sensors and fluctuating network traffic, ensuring detection capabilities and security infrastructure remain intact. Similarly, Naeini et al. [82] implement an adaptive controller that selects features and operational modes based on sensor availability and signal quality, optimizing sensor use and dynamic model configuration. Both solutions highlight the importance of flexibility in sensor and data management within IoMT systems, demonstrating that adaptability is crucial for ensuring resilience and reliability.

##### Failure Handling

Failure handling is another critical aspect of technological adaptability. IoMT systems must be designed to adjust their operations in case of component or device failures. In such scenarios, systems need to ensure continuity despite failures in nodes or devices. In that direction, Gupta et al. [83] propose a federated learning framework that addresses node failures by redistributing models among nearby edge nodes when a computing node cannot perform local training. This decentralized approach ensures system operability, maintaining performance and data integrity without disruption. It highlights the significance of redundancy and distributed processing in handling failures, guaranteeing continuous service in critical healthcare applications where reliability is crucial.

##### Resource Optimization

Resource optimization focuses on the system’s ability to dynamically optimize resource usage to maintain efficiency. IoMT systems must adjust their operations based on available resources to minimize energy consumption, reduce latency, and balance network loads. Several strategies have been proposed in the literature, all aiming to ensure efficient use of computational and network resources. For example, Alatoun et al. [20] propose a solution where tasks are dynamically assigned to computing nodes based on the available resources, optimizing energy consumption, latency, and network usage. This dynamic task allocation ensures that the system operates at peak efficiency, responding to real-time variations in resource availability. A complementary approach by Almudayni et al. [22] employs a fuzzy system that dynamically allocates resources and prioritizes tasks based on the computing capacity of the system’s nodes. By factoring in the capabilities of each node, this system ensures that resources are utilized optimally, further improving the overall efficiency of the system. In the domain of federated learning, Zhang et al. [84] propose a framework featuring a device selection module, which uses deep reinforcement learning to identify the optimal local update strategy for each edge device. This module effectively minimizes the energy cost for edge devices, demonstrating how machine learning techniques can enhance resource allocation. Iftikhar et al. [85] introduce a fog broker node that assigns tasks to fog computing nodes based on a calculated availability index, ensuring that tasks are distributed according to current resource conditions. This approach leverages the fog computing architecture to enhance resource management and operational efficiency. Rajpoot et al. [23] employ an Ant Colony Optimization algorithm for load balancing, a biologically inspired approach designed to optimize resource usage by distributing tasks more effectively across the network. Similarly, Yadav et al. [21] utilize reinforcement learning for dynamic task offloading, reducing energy consumption and latency by making real-time decisions about where tasks should be executed.

Collectively, these approaches underscore the importance of adaptive resource management in IoMT systems. Whether through dynamic task allocation, machine learning techniques, or optimization algorithms, each solution seeks to maximize system efficiency while minimizing resource waste, ensuring that IoMT systems can operate smoothly in resource-constrained environments.

#### 5.4.2. Clinical Needs Runtime Adaptation

In the context of IoMT systems, adaptability to patient conditions is a crucial feature that ensures real-time, personalized healthcare. This adaptability can be categorized into three main areas: *health condition*, *treatment*, and *clinical needs*, with several solutions in the literature addressing these aspects.

##### Health Condition Adaptability

Health condition adaptability means that systems must adjust their operations dynamically based on the real-time health status of the patient. Alatoun et al. [82] propose a solution where tasks are dynamically offloaded based on the patient’s condition, ensuring optimal resource allocation in response to patient health. Similarly, Bui et al. [56] develop a system that triggers a survey whenever an event of interest related to the patient’s health occurs, providing timely data collection tailored to the individual’s condition. Jazaeri et al. [67] introduce an edge computing process that prioritizes resources for patients with abnormal vital signs, improving caching and data transmission to ensure fast responses for those in critical need. Almudayni et al. [24] propose a fuzzy system that allocates resources and prioritizes tasks based on estimated patient conditions, optimizing system performance while ensuring patient safety. Furthermore, Zida et al. [86] develop a risk assessment feature that identifies patients who require further investigation and ensures that urgent data is processed at the cloud level with priority. Panja et al. [28] also provide a mobile app that performs on-demand monitoring for high-risk patients, responding immediately to critical health status changes.

##### Treatment Adaptability

Treatment adaptability means that systems suggest personalized recommendations and treatment plans autonomously. Elbagoury et al. [19] propose a mobile AI agent that leverages explainable AI to deliver personalized surveillance, dynamically suggesting prescribed procedures and active treatment recommendations. Tsiouris et al. [40] propose a system that uses reinforcement learning to autonomously recommend and personalize weekly treatment plans according to the patient’s evolving needs. Hameed et al. [87] further develop a rule inference engine capable of matching patient symptoms to knowledge records, thereby suggesting relevant treatments in real-time for various diseases.

##### Clinical Needs Adaptability

Clinical needs adaptability addresses the system’s ability to adjust its operations based on the requirements of healthcare providers or institutions. Elghoul et al. [88] propose a mobile app with dual operational modes for data acquisition, which adjusts its behavior depending on whether the patient is in a hospital or home setting. Additionally, another system by Chen et al. [89] adapts its data collection based on the clinician’s directives and patient responses to questionnaires, ensuring the data collected are aligned with clinical priorities.

These solutions across health condition, treatment, and clinical needs highlight the critical role of adaptability in IoMT systems, ensuring personalized, real-time responses to the evolving needs of patients and healthcare providers.

Table 5 categorizes the self-adaptation strategies found in the reviewed literature according to their scope, technological or clinical. Each row describes a specific adaptation dimension, along with the techniques employed and representative references.

### 5.5. RQ5: Customizability

The customization of IoMT systems to meet unique clinical needs and technological environments implies an analysis of their configuration processes. Specifically, this section addresses the ability of such systems to integrate diverse sensors by leveraging *interoperability standards*, *communication protocols*, and *device-independent technologies*.

#### 5.5.1. Interoperability Standards

Solutions based on interoperability standards aim to facilitate seamless integration of heterogeneous sensors by adopting widely recognized frameworks. For instance, Jameel et al. [71] propose a solution utilizing the OpenEHR standard and a technical domain ontology. This approach enables the identification of sensors and automatic translation of sensed data, ensuring interoperability and standard-compliant data management.

#### 5.5.2. Communication Protocols

Another class of solutions relies on implementing standardized communication protocols to enable integration across a broad spectrum of devices. Several notable contributions fall within this category. For example, Tsiouris et al. [13] introduce an IoT device management module that integrates various devices through standardized hardware–software communication protocols such as Bluetooth Low Energy (BLE), Bluetooth, Wi-Fi, and USB. Similarly, Said et al. [79] propose a system employing an MQTT broker to decouple sensors from the applications processing the collected data, enhancing modularity and scalability. Sriborrirux et al. [38] propose a microservices-based central hub using BLE to facilitate smooth interaction with a diverse range of devices.

#### 5.5.3. Device-Independent Technologies

Solutions leveraging device-independent technologies prioritize functionality across diverse platforms and architectures, ensuring flexibility and adaptability. For instance, Elbagoury et al. [90] present a mobile agent capable of receiving inputs from various medical sensors, with a particular focus on electromyography monitoring. Similarly, Emish et al. [27] design a mobile application using React frameworks to retrieve GPS and motion data, designed to function independently of specific mobile platforms. Building on this approach, Naeini et al. [82] propose a system for multimodal data acquisition that imposes no restrictions on the types of devices used, highlighting its versatility and adaptability to different technological environments.

Table 6 summarizes the main strategies adopted in the reviewed literature to enhance the customizability of IoMT systems.

### 5.6. Cross-Cutting Contributions Across Non-Functional Requirements

**Figure 4:**
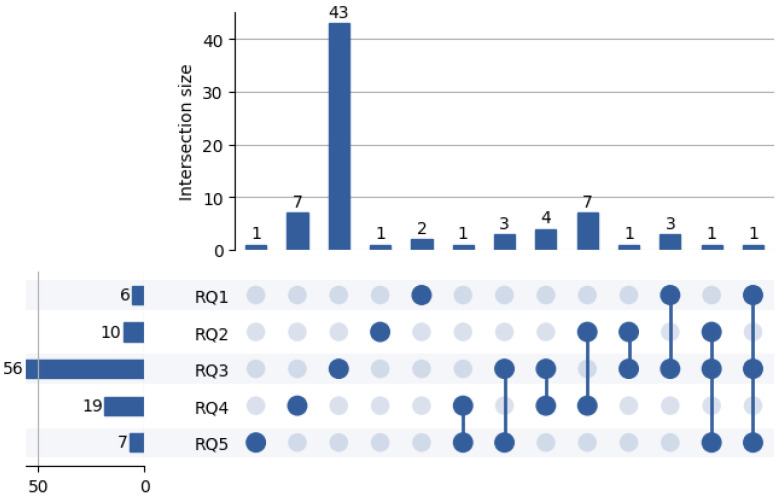
UpSet plot showing the intersection of non-functional requirements addressed by the reviewed studies. The horizontal bars on the left indicate the total number of articles that address each individual research question. The vertical bars represent the number of studies covering the combinations of RQs indicated by the connected dots below. This visualization highlights how some works focus on a single requirement (e.g., only security), while others contribute to multiple non-functional aspects simultaneously.

To better understand the overlap across non-functional requirements addressed in the reviewed literature, we analyzed how individual studies contribute to multiple RQs. Figure 4 presents an UpSet plot summarizing these intersections. As shown, while a substantial number of papers focus on a single requirement, a noteworthy portion addresses two or more aspects concurrently.

## 6. Findings and Future Research

This review highlights that IoMT systems have achieved significant advancements, particularly in terms of technological innovation and system integration. However, critical gaps remain in addressing non-functional aspects such as interoperability, sustainability, security, runtime self-adaptation, and configurability. These aspects are vital for ensuring that IoMT solutions not only meet immediate clinical needs but also maintain long-term operability and adaptability in dynamic healthcare environments.

Key insights from the analysis reveal the need for a more comprehensive understanding of how IoMT systems can ensure seamless communication across heterogeneous platforms, optimize resource usage for sustainability, and protect sensitive medical data without compromising accessibility. Furthermore, adaptability in real-time to technological constraints and patient-specific requirements remains partially underexplored, despite its importance for personalized and efficient care delivery. This study highlights these findings, emphasizing the importance of further investigation in these areas.

### 6.1. Interoperability

Interoperability is central to IoMT systems, enabling seamless communication between heterogeneous devices and platforms. Frameworks such as OpenEHR and FHIR have been widely adopted to standardize data exchange and enhance system compatibility [13,14].

However, variations in their application across solutions highlight a gap in achieving universal interoperability. Additionally, integrating legacy systems with newer technologies remains a challenge, limiting the scalability and effectiveness of IoMT solutions in real-world healthcare environments.

Given the current challenges, future research should focus on developing universal frameworks that effectively combine both syntactic and semantic interoperability standards. By addressing these two dimensions together, it will be possible to enable more comprehensive and efficient data exchange across diverse systems and platforms, ultimately improving the integration and usability of IoMT solutions.

### 6.2. Sustainability

IoMT systems often face constraints related to energy consumption and resource utilization. Distributed computing architectures, such as fog and mist computing, play a significant role in reducing latency and improving energy efficiency [19,20,22].

However, most solutions fail to align with *green computing goals*, which are crucial for the sustainability of IoMT deployments. While optimizing energy consumption and resource use is common, there is limited focus on practices such as device reuse, recycling, and lifecycle management. These aspects, along with reducing the carbon footprint of production and transportation, are essential for ensuring that IoMT systems are not only energy-efficient but also environmentally sustainable over time.

Future research should focus on leveraging AI-driven optimization techniques to dynamically manage resource allocation in IoMT systems. By focusing on reducing energy consumption and minimizing environmental impact, these techniques could significantly improve the sustainability of IoMT solutions, ensuring they are both efficient and eco-friendly in real-world healthcare environments.

### 6.3. Security

The analysis reveals that ensuring the CIA triad (confidentiality, integrity, and availability) is paramount for IoMT systems. Blockchain technology has emerged as a robust mechanism for securing medical data through its decentralized and tamper-resistant architecture [16,43]. Additionally, advanced encryption techniques such as elliptic curve cryptography [35] and homomorphic encryption [34] have been employed to protect sensitive information.

Despite these advancements, a key challenge remains in developing lightweight, scalable security solutions for resource-constrained IoMT devices, such as wearable sensors. These devices often have limited processing power, memory, and battery life, making traditional security mechanisms difficult to implement without affecting performance. Thus, there is a need for novel security architectures that balance strong protection with the unique constraints of IoMT devices, ensuring secure data transmission and storage without compromising functionality.

Future research should focus on designing lightweight and scalable encryption mechanisms tailored for resource-constrained IoMT environments. By addressing the unique limitations of IoMT devices, such as limited processing power and memory, these encryption solutions could enhance data security without compromising performance, enabling secure communication and data storage in diverse healthcare settings.

### 6.4. Runtime Self-Adaptation

Adaptability mechanisms, such as failure handling, resource optimization, and sensor adaptability, are crucial for maintaining system performance under varying conditions [82,83].

Despite these advances, predictive mechanisms that proactively address potential issues, such as sensor malfunctions or network constraints, should be better analyzed. Current adaptation strategies tend to be reactive, addressing problems as they arise, but there is a need for systems that can anticipate and mitigate issues before they affect performance. For instance, predicting sensor failures or network congestion in advance could allow the system to reallocate resources or adjust operations proactively, maintaining system reliability and performance. Integrating such predictive models would significantly enhance the robustness of IoMT systems in dynamic healthcare environments.

Future research should focus on developing predictive adaptation mechanisms that proactively address potential issues such as sensor failures, network constraints, and evolving patient needs. By anticipating these challenges before they impact system performance, IoMT solutions could adapt in real-time to ensure continuous, reliable operation, enhancing both patient care and system efficiency in dynamic healthcare environments.

### 6.5. Configurability

Device-independent technologies have shown promise in enabling IoMT systems to operate across diverse platforms, architectures, and clinical contexts. Solutions such as mobile agents [8] and multimodal acquisition systems [82] demonstrate flexibility and adaptability.

However, their scalability and configurability within complex healthcare infrastructures require further investigation. While these device-independent technologies demonstrate flexibility in supporting interoperability across diverse platforms and clinical contexts, ensuring their efficient configuration for different healthcare environments remains a challenge. Key issues include the ability to adapt to different system requirements, workflows, and device specifications, as well as to dynamically adjust to evolving healthcare needs. Research is needed to develop methods for configuring these solutions to integrate seamlessly with existing systems, such as electronic health records (EHRs) and hospital information systems (HIS), while maintaining performance, security, and usability in diverse, dynamic, and increasingly complex healthcare environments.

Future research should focus on designing modular, device-agnostic architectures that simplify the customization of IoMT systems for diverse healthcare contexts. By enabling seamless adaptation to different devices, workflows, and clinical needs, these architectures could enhance the flexibility and scalability of IoMT solutions, ensuring they are easily configurable to meet the specific requirements of various healthcare environments.

### 6.6. Summary of Research Questions and Key Findings

To improve the readability of the findings and provide a concise overview of the results obtained from the systematic review, Table 7 summarizes how each research question (RQ1–RQ5) has been addressed. For each RQ, the table highlights the key aspects investigated, the number of supporting studies, representative techniques, and a synthesized conclusion. This summary complements the detailed discussion provided earlier and supports a clearer understanding of the contributions and limitations identified across the literature.

## 7. Threats to Validity and Critical Insights

Although this review followed a rigorous and systematic methodology, certain limitations may affect the validity and generalizability of the conclusions drawn. In this section, we discuss the main factors that may have influenced the interpretation of the evidence.

First, our findings are inherently dependent on the quality and completeness of the reviewed articles. In several cases, essential information was missing (e.g., implementation details or evaluation methodologies), which may have hindered a deeper assessment of how non-functional requirements were addressed in practice. As a result, some conclusions may rely on partial or inferred evidence.

Second, the classification and analysis process inevitably involve a degree of subjectivity. Despite relying on established definitions and applying a consistent methodology, the mapping between proposed solutions and quality attributes is not always unambiguous. Concepts such as “configurability” or “interoperability” may be interpreted differently across studies, which may have affected our synthesis.

Third, IoMT applications are highly context-sensitive. Variability in healthcare infrastructures, regulatory frameworks, and technological maturity across environments may limit the uniform applicability of our conclusions. For example, recommendations on system integration or energy optimization may be more relevant in large-scale hospital systems than in rural or resource-limited settings.

Finally, despite a comprehensive search strategy and defined inclusion criteria, the risk of selection bias remains. Relevant studies might have been unintentionally excluded due to differing terminology, limited indexing, or publication outside the selected venues. This may have impacted the completeness of the evidence base and the robustness of our synthesis.

Beyond these methodological considerations, we also identified notable trends and limitations in the studies themselves. Only 5 works validated their solutions in real-world deployments, while 16 used real sensor data, and 22 employed existing datasets in simulated scenarios. Twenty-one studies relied entirely on synthetic simulations, and 12 lacked any information about implementation or evaluation. These numbers highlight a general lack of empirical validation, which may limit the practical generalizability of proposed solutions.

In addition, 26 of the 56 security-focused studies adopted blockchain technologies, using them for tasks such as data storage and access control via smart contracts. However, few of these works discussed the computational or energy overhead associated with blockchain—a key consideration for resource-constrained IoMT environments. This suggests a tendency to prioritize theoretical benefits without fully addressing implementation feasibility. Future research should seek to evaluate not only the effectiveness but also the operational costs of such solutions.

Despite the methodological threats discussed and the limitations identified within the reviewed studies, we believe this review provides a valuable and timely contribution to the field. By synthesizing a diverse body of work and critically reflecting on current practices, the review offers meaningful insights into how non-functional requirements are addressed in IoMT systems. The identification of key gaps and recurring challenges lays a solid foundation for future investigations. These insights can support both researchers and practitioners in advancing the design, deployment, and optimization of IoMT solutions, ultimately contributing to the development of more intelligent, resilient, and sustainable digital healthcare infrastructures.

## Figures and Tables

**Table 1:** Summary of RQ1: Interoperability technologies.

	Standards			APIs		
	Open mHealth	FHIR	OpenEHR	REST API	Blockchain API	Tracking Platform API
**Standards for data formatting**	[15]	[13]	[14]			
**Integration of third-party systems**				[13]		[13,15]
**Exposure to third-party systems**				[13,14]	[16,17,18]	

**Table 2:** Summary of RQ2: Sustainability strategies and resource optimization techniques in IoMT systems.

	Distribution Algorithms	Event-Based Algorithm	Optimization Algorithms	Distributed Computing
**Delay**	[23]	[19]	[23]	[19,23]
**Latency**	[20,21,22]		[21,24,25]	[20,22]
**Processing**	[22,23]			[22]
**CPU usage**	[20]	[26]		
**Network usage**	[20]	[26,27,28]	[24]	[20]
**Storage usage**		[26]	[24]	

**Table 3:** Summary of RQ3: Techniques used to ensure the confidentiality and integrity of the CIA triad.

Features	Techinques	Approach	References
**Authentication**	Digital Signature	Elliptic Curve-based	[16,32,33,34,35,36,37] (with smart contracts)
			[38]
		Attribute-based	[39,40] (with smart contracts)
		Asymmetric	[18] (with smart contracts)
		Not specified	[41]
	Digital Signature-based Certificates	Elliptic Curve Signature Certificate	[42] (with smart contracts)
		Elliptic Curve Qu Vaston Certificate	[17] (with smart contracts)
		Not specified	[43] (with smart contracts)
	Identity-based	Credentials	[44,45,46,47,48] (with smart contracts)
			[13,25,49,50,51]
	Multi-factor		[35,41,52,53]
	Physical Unclonable Function		[32,54]
	Authentication scheme	Pseudonym-based	[55]
		Elliptic Curve Cryptography-based	[56]
		Identity-based	[57,58]
		Zero Knowledge Proofs	[59]
**Access Control**	Digital Signature	Elliptic Curve-based	[16,35,58] (with smart contracts)
		Attribute-based	[39,40] (with smart contracts)
	Identity-based	Identity relationships	[17,45] (with smart contracts)
			[49]
	Role-based		[18,36,41,47,48,60] (with smart contracts)
			[33,50]
	Rule-based		[43,46] (with smart contracts)
	Others	Triple-subject purpose-based	[61] (with smart contracts)
		Selective-ring-based	[37] (with smart contracts)
		Access logging	[62] (with smart contracts)
		Approval-based multi secret sharing	[51]
**Encryption**	Symmetric	Lightweight algorithm	[63]
		Not specified	[36,41] (in blockchain)
			[17,41,53,56]
	Asymmetric	Homomorphic encryption	[16,18,34,40,62] (in blockchain)
			[39,63,64,65,66]
		Elliptic Curve encryption	[35,37,42,44,58] (in blockchain)
			[33,35,37,42,44,67]
		Attribute-based encryption	[68]
		Not specified	[47]
	Others	Secure Multi-Party Computation	[16] (in blockchain)
			[69]
		Identity-based	[57]
		Sparse Learning-based	[70]
		Certificateless proxy re-encryption	[55]
		SSL protocol encryption	[27,71]
		Not specified	[43] (in blockchain)
			[46]

**Table 4:** Summary of RQ3: Techniques used to ensure the availability of the CIA triad.

Features	Techinques	Approach	References
**Attack detection**	Machine Learning	Incremental classifier	[72] (fog layer)
**Intrusion detection**	Deep Learning	Deep Neural Network	[73,74] (edge layer)
		Convolutional Neural Network	[75] (edge layer)
		Not specified	[57] (cloud layer)
	Machine Learning	Long Short Term Memory	[75] (edge layer)
	Federated Learning	Long Short Term Memory	[76]
		Support Vector Machine	[46]
		Random Forest	[46]
		Reinforcement Learning	[77]
		Model independent	[78]
**Anomaly detection**	Machine Learning	Support Vector Machine	[79] (edge layer)
		Model independent	[80] (edge layer)
	Probabilistic Model	Gaussian Mixture Model	[34] (edge layer)
	Stochastic Model	Hidden Markov Model	[81] (cloud layer)

**Table 5:** Summary of RQ4: Dimensions of self-adaptation in IoMT systems.

Adaptation Category	Adaptation Dimension	Approach	References
Technological runtime adaptability	Sensor and data adaptability	Adaptive controllers, fog-based systems	[72,82]
	Failure handling	Federated learning, edge redistribution	[83]
	Resource optimization	Task offloading, fuzzy systems, reinforcement learning, optimization algorithms	[20,21,22,23,84,85]
Clinical Runtime Adaptation	Health Condition Adaptation	Task offloading, vital sign prioritization, risk-based routing	[24,28,56,67,82,86]
	Treatment Adaptation	Explainable AI, reinforcement learning, rule inference engines	[19,40,87]
	Clinical Needs Adaptation	Mode switching, clinician-driven data adaptation	[88,89]

**Table 6:** Summary of RQ5: Customizability strategies in IoMT systems.

Customization Strategy	Approaches	References
Interoperability standards	Standardized data models, domain ontologies	[71]
Communication protocols	BLE, Wi-Fi, USB, MQTT, microservices-based hubs	[13,38,79]
Device-independent technologies	Mobile agents, cross-platform apps, multimodal data acquisition	[27,82,90]

**Table 7:** Summary of research questions, addressed areas, and insights.

Research Question	Key Technological Focus	# of Studies	Main Insights and Open Issues
**RQ1: Interoperability**	FHIR, OpenEHR, REST APIs, Blockchain APIs, semantic data models	6	Standard adoption is inconsistent and fragmented; semantic interoperability and integration with legacy healthcare systems remain significant challenges. Efforts are needed to unify standards and facilitate widespread adoption.
**RQ2: Sustainability**	Task offloading, fog/mist computing, event-based sensing, optimization algorithms, lifecycle strategies	10	Energy efficiency is commonly addressed through optimized computational offloading and fog/mist computing. However, lifecycle management practices, environmental impact quantification, and full-lifecycle sustainability assessments remain limited and require further attention.
**RQ3-CI: Confidentiality & Integrity**	Encryption (ECC, homomorphic), access control, blockchain-based authentication	46	Many approaches effectively secure data during storage and transmission using strong encryption and blockchain technologies. Nevertheless, lightweight encryption tailored to resource-constrained IoMT devices is underexplored, and trust management integrated with clinical workflows requires deeper investigation.
**RQ3-A: Availability**	Intrusion/anomaly detection, federated learning	13	Availability is primarily ensured through anomaly detection and federated learning techniques. However, empirical validation in real-world scenarios is sparse, and potential energy-performance trade-offs are frequently overlooked, highlighting a gap in practical evaluation frameworks.
**RQ4: Self-Adaptation**	Failure handling, adaptive sensing, reinforcement learning	19	Existing self-adaptation solutions predominantly adopt reactive approaches addressing immediate technological failures or clinical variations. Predictive and proactive adaptation strategies that anticipate changes and issues remain insufficiently explored and represent a significant research opportunity.
**RQ5: Configurability**	Device-agnostic frameworks, technical domain ontologies, custom acquisition systems	7	While configurability is recognized, there is limited advancement in developing modular, reusable architectures explicitly designed for diverse and evolving healthcare scenarios. Future research should focus on enhancing system modularity and integration capabilities.

## Data Availability

No new data were created or analyzed in this study. Data sharing is not applicable to this article.
